# Neuroscience in the third dimension: shedding new light on the brain with tissue clearing

**DOI:** 10.1186/s13041-017-0314-y

**Published:** 2017-07-20

**Authors:** Robin J. Vigouroux, Morgane Belle, Alain Chédotal

**Affiliations:** Sorbonne Universités, UPMC Univ Paris 06, INSERM, CNRS, Institut de la Vision, 17 Rue Moreau, 75012 Paris, France

**Keywords:** 3DISCO, iDISCO, Clarity, Tissue-clearing, Neuroscience

## Abstract

For centuries analyses of tissues have depended on sectioning methods. Recent developments of tissue clearing techniques have now opened a segway from studying tissues in 2 dimensions to 3 dimensions. This particular advantage echoes heavily in the field of neuroscience, where in the last several years there has been an active shift towards understanding the complex orchestration of neural circuits. In the past five years, many tissue-clearing protocols have spawned. This is due to varying strength of each clearing protocol to specific applications. However, two main protocols have shown their applicability to a vast number of applications and thus are exponentially being used by a growing number of laboratories. In this review, we focus specifically on two major tissue-clearing method families, derived from the 3DISCO and the CLARITY clearing protocols. Moreover, we provide a “hands-on” description of each tissue clearing protocol and the steps to look out for when deciding to choose a specific tissue clearing protocol. Lastly, we provide perspectives for the development of tissue clearing protocols into the research community in the fields of embryology and cancer.

## Introduction

Over the past century, biological sciences have evolved from a physiological to a cellular and then a molecular understanding. In the forefront of biological sciences, novel imaging techniques have increased our ability to image biological samples to molecular resolutions through the development of two-photon microscopy, stimulated emission depletion microscopy (STED) and stochastic optical reconstruction microscopy (STORM) amongst others. Although these techniques allow high resolution, the depth at which they can image tissues is limited and as a result constrains our analysis to small volume samples or histological sections.

In recent years there has been an active shift from imaging thin samples to whole-organ, and more recently whole mammalian animals thanks to novel tissue clearing protocols. The notion of tissue clearing took birth over 100 years ago through the work of Spalteholz [1, 2]. It stemmed from an anatomical perspective to fully understand how an organ worked in its “natural” 3 dimensional (3D) form rather than in two-dimensions. Researchers quickly understood that to observe an organ in 3D they had to: i) minimize light artifacts (scattering and absorption) from the tissue, and ii) optimize microscopy techniques to achieve high resolution.

Light absorption is an inherent property of tissues due to their different chemical composition (water, proteins and lipids), which result in a heterogeneous refractive index. The key to reduce light absorption is to homogenize the refractive index of the sample by removing lipids and subsequently immersing it into a medium with a refractive index matching that of the fixed proteins in the sample. This achieves an equilibration of both intra- and extra-cellular refractive indices thereby rendering the tissue “transparent”.

The quest for the ideal agent capable of homogenizing the refractive indices of tissues took a burst in early 2000’s. Tissue clearing protocols have since diversified into over 10 different clearing agents. The diversity of clearing protocols is beyond the scope of this article, for a comprehensive discussion on this topic you can refer to the following recent reviews [3–6]. What has become apparent in the past five years is that the field has split into two main approaches: i) solvent-based clearing, and ii) aqueous-based clearing techniques.

In this review, we aim to focus on 3D imaging of solvent-cleared organs (3DISCO) and the Clear Lipid-exchanged Acrylamide-hybridized Rigid Imaging/immunostaining/in situ hybridization-compatible Tissue hYdrogel (CLARITY)-based clearing protocols. We investigate the recent optimizations of each technique and propose a guideline for choosing the appropriate protocol for your specific scientific question.

## Clearing strategies

### The organic approach

Since Spalteholz’s clearing procedure, using a mixture of methyl salicylate/benzyl benzoate and wintergreen oil [1, 2], three main limitations have persisted in solvent-based clearing protocols: i) tissue transparency, ii) immuno-labeling, and iii) endogenous fluorescence stability. The search for the ideal combination of solvents took a turn in the early 2000’s, Dodt et al. proposed a novel combination of ethanol and hexane de-hydration followed by an immersion in Benzyl Alcohol/Benzyl Benzoate (BABB) for clearing tissues [7]. This method was efficient for clearing tissues and for the first time could be coupled to Light Sheet Fluorescence Microscopy (LSFM), to image centimeter-sized samples. Whilst this method allowed the visualization of endogenous fluorescence and was amenable to immuno-labelling, the authors noted that this procedure could not fully clear adult tissues and that fluorescence was rapidly quenched (within a few hours).

To overcome these limitations, in 2012 Ertürk et al. developed a clearing protocol named 3DISCO. Instead of alcohol, the authors proposed tetrahydrofuran (THF). Whilst being a strong de-hydrating agent, THF is also a very potent de-lipidating agent. Thus, enhancing the homogeneity of the samples’ refractive index. This is coupled with a supplementary de-lipidation treatment in dichloromethane (DCM) and an immersion in dibenzyl ether (DBE) as the imaging medium. The authors show that unlike the BABB protocol, they could efficiently clear entire adult tissues while maintaining the analysis of proteins using either endogenous fluorescence or immuno-labelling [8–10]. In addition, they showed that 3DISCO increased the signal to noise ratio while prolonging the integrity of the fluorescence. Furthermore, this protocol could be applied to a vast array of tissues ranging from lipid-rich structures such as the brain, but also extracellular matrix-rich structures such as gingiva [6]. Overall, 3DISCO offered a standardized protocol for clearing multiple tissues in a simple, efficient and cost-effective manner that was widely applicable.

A hallmark of 3DISCO-clearing is the resulting tissue-shrinkage (Table 1). In an attempt to prevent this, Renier and colleagues developed a novel clearing protocol solely based on methanol de-hydration, the immunolabeling-enabled three-dimensional imaging of solvent-cleared organs (iDISCO+) [11]. The steadily increasing methanol concentrations result in modest tissue-shrinkage (about 10%). In addition, the “transparency” of an adult mouse brain is highly increased. Making use of this mild tissue shrinkage, Renier et al. have developed a computational pipeline for iDISCO + −cleared brains to precisely analyze axonal tracts and nuclei in respect to their anatomical location, called Clearmap.

**Table 1:** A hands-on comparison of tissue clearing protocols

	Mouse Stages			Immuno-staining		Clearing	Endogenous Fluorescence	Tissue shrinkage	Clearing Performance	Cost	Toxicity	
	<E15	E15-P0	Adult	Before Clearing	After Clearing						Clearing Solutions	Imaging Medium
Solvents												
3DISCO	Entire embryo	Isolated organs	Isolated organs	1–3 wks	−	1 d	−	+++ (up to 50%)	+++	+	+++	+
iDISCO+	Entire embryo	Entire embryo	Isolated organs	2 wks	−	2 d	−	+ (up to 10%)	+++	+	+++	+
uDISCO	−	−	Entire animal	2 wks	−	3 d - 1 wk	Yes (up to 4 wks)	+++ (up to 40%)	+++	+	+++	+++
Reagents												
CLARITY	Entire embryo	Entire embryo	Isolated organs	−	1–3 wks	5 d - 2 m	Yes	*	++	+++	+++	−
PACT	−	−	3 mm slice	−	1–2 wks	2 wks	Yes (up to 2 m)	*	++	++	+++	−
PARS	−	−	Entire animal	−	10 d	4 d - 2 wks	Yes (up to 3 m)	*	++	++	+++	−

A comparison of most used clearing protocols. – = no information/ not applicable; + = short/low; +++ = long/high; * = varies between mounting media

Nonetheless, there remained the issue of endogenous fluorescence stability. To this end, Pan et al. designed a novel clearing protocol, ultimate-3DISCO (uDISCO) [12]. Firstly, using *tert*-butanol as a more stable de-hydrating agent, the tissue is then treated with DCM as in prior protocols. Finally, the authors immerse the sample in an imaging medium composed of di-phenyl ether (DPE)/BABB and the anti-oxidant alpha-tocopherol (vitamin E). This imaging medium, renamed BABB-DA, was identified following a screen of organic compounds to promote both the stability of proteins and maintain high tissue transparency. Endogenous fluorescence and tissue transparency are at two ends of the spectrum. Pan et al. show that the uDISCO protocol can fluctuate between higher transparency and better endogenous fluorescence integrity depending on the ratio of DPE to BABB. Indeed, this is applied by either carrying whole-body clearing (using a high DPE ratio) or brain clearing (using a low DPE ratio). However, tuning the appropriate ratio of DPE to BABB for individual tissues to balance between endogenous fluorescence and tissue transparency is an important drawback with this protocol. Furthermore, to achieve whole-body clearing, the authors carry out a tedious protocol that requires perfusing mice for several days to weeks using BABB-DA. Together, the applicability of using uDISCO on a large scale will be quite challenging until further optimizations to the protocol are made.

### The detergent approach

In 2011, Hama et al. proposed for the first time the possibility to clear tissues using an aqueous-based approach, Scale [13]. The authors showed that by using concentrated urea in an aqueous solution they could efficiently render tissue transparent. However, the harsh conditions of clearing led to significant protein loss (~41%), which brought severe limitations to studying the tissue following clearing [14].

To limit damage while maintaining high tissue transparency, Chung et al. developed in 2013 an aqueous-based clearing technique, termed CLARITY [14–16]. In principle, CLARITY consists of infusing the sample with hydrogel monomers together with temperature-sensitive polymerization initiators. Upon heating, the hydrogel polymerizes creating covalent bonds to proteins as well as taking the shape of the tissue. To clear the tissues rapidly, the authors remove lipids by bathing the sample in an ionic detergent solution coupled to an electrophoretic machine. Finally, the sample is immersed in an imaging medium. Recently, a lot of work has been done to expand the repertoire of imaging media compatible for CLARITY-cleared samples (FocusClear, RIMS, RapidClear, Diatrizoate-derived), these all contain variations of resonant rings and iodine complexes. Of note, depending on the immersion medium, the sample can either expand, shrink or maintain its initial size [17]. Alterations to this protocol are constantly updated; you can refer to the online platform: forum.claritytechniques.org. However, many aspects of CLARITY hinder its usage. Firstly, the expense of the materials from the electrophoretic tissue clearing machines to the FocusClear immersion agent (Table 1). In addition, Electrophoretic tissue clearing (active CLARITY) is relatively efficient (~5 days for an adult mouse brain) but can result in tissue degradation/distortion due to heat production by the electrophoretic machine. Finally, whilst the passive CLARITY protocol avoids this tissue perturbation, the protocol is incredibly long (~2 months for an adult mouse brain) [14, 16].

The limiting factor in the process of tissue clearing is the de-lipidation step. To address this, Yang et al. proposed an optimized passive CLARITY protocol, named passive CLARITY technique (PACT) [18]. By altering the hydrogel matrix, the authors were able to increase (passively) the rate of de-lipidation of the sample in a bath of ionic detergent, from months to 12 days for a 3 mm adult mouse brain block. PACT was shown to be suitable with immunohistochemistry and for the first time could be amenable to small-molecule fluorescent in situ hybridization (smFISH), to visualize mRNA transcripts [18]. Whilst increasing the rate of tissue clearing, Yang et al. only carry out the PACT protocol on 1-3 mm tissue blocks and not on entire organs. Indeed, the smFISH immuno-labelling was only carried out on 100 μm-thick brain slices. Furthermore, by reducing the acrylamide content of the hydrogel matrix the cleared samples are extremely fragile, which can significantly hinder the imaging potential of the tissue.

To increase the rate of de-lipidation further, the authors described an alternate protocol named perfusion assisted agent release in situ (PARS) [18]. In contrast to bathing the sample in the ionic detergent, PARS consists in continuous perfusion of clearing agents either by intra-cardiac or intra-cerebrospinal administration. Here, the authors attain a modest clearing by 4 days for an adult mouse brain to 2 weeks for an adult mouse spinal cord (Table 1). However, reproducibility of tissue clearing between animals using this method is a significant issue. Indeed, obstruction of major vessels could dramatically hinder the clearing of the tissues. In addition, the complexity of the perfusion procedure limits ones ability to screen a large number of animals at once.

Recently, an enhanced CLARITY protocol was developed. This protocol made use of both PACT and PARS protocols by reducing the acrylamide percentage and by circulating an SDS-rich solution to increase the de-lipidation rate. The authors report clearing an adult mouse brain in 12 days [19]. This is a great improvement of passive techniques, however, owing to the low acrylamide mesh, the fragility of the samples remains a major issue.

## Visualizing your sample

The vast catalogue of tissue-clearing protocols published attest to the diversity of requirements from the scientific community. The purpose of clearing any tissue is to analyze a specific cell type and its distribution within a tissue of interest. This goal can be achieved by using a transgenic reporter, a viral infection, or by antibody labeling. In this section, we will describe the limitations and advantages of solvent-based or aqueous-based clearing methods in answering each of these three questions.

### Immuno-fluorescence

Basic histological staining and immuno-histochemistry remain a central part of all research areas. However, immuno-stained tissues have for decades relied on thin sections and therefore a 2-dimensional observation, which inevitably provided only partial information in regards to the organization of a tissue. One striking example of this is portrayed by mouse retinal ganglion cells of the retina that extend in various directions to reach several brain targets which can span several millimeters apart [20]. To date, research groups have adapted immuno-labelling using IgG, Fab, and nanobodies ranging from 14KDa to 150KDa respectively [21, 22]. However, since most assays commonly use IgG antibodies, our discussion will focus on these.

Initially, aqueous-based protocols, such as CLARITY, provided a strong platform for immuno-labelling compatibility. Indeed, Chung et al. showed that total protein loss following 0.1% TritonX-100 treatment of paraformaldehyde fixed tissues was comparable to protein loss following CLARITY tissue clearing (24 and 8%, respectively) [14]. Whilst the authors showed CLARITY was amenable to immuno-labelling, the majority of their molecular phenotyping rested on 1 mm-thick brain blocks and only a single antibody, Tyrosine Hydroxylase, was validated for whole-brain immuno-labeling. Another strength of the CLARITY protocol was the possibility for multiple rounds of immuno-labelling by eluting previously cross-linked antibodies. To our knowledge, until this day, only the developers of the Clarity protocol have shown multiple-round immuno-labelling on 1 mm-thick brain blocks using five antibodies (tyrosine hydroxylase, parvalbumin, glial fribrillary acidic protein (GFAP), choline acetyltransferase (ChAT), and DAPI) [14, 16]. Of note, the impact of several rounds of elution on tissues remains to be investigated, particularly whether certain epitopes could be affected in a heterogeneous manner and thus result in tissue labeling variability.

Nonetheless, one major limitation associated with CLARITY remains the diffusion of antibodies through the hydrogel matrix which depends on simple diffusion of antibodies and is a notoriously lengthy process (up to 6 weeks for an adult mouse brain) [15]. Since, derivatives of CLARITY have provided altered immuno-staining protocols by altering the hydrogel matrix which only provided a modest increase in the rate of labeling but consisted of constant antibody replenishing (every 3 days) and large volumes resulting in incredibly costly procedures [18, 21].

To optimize immuno-staining of whole-tissue, several groups have since developed a variety of protocols. The overall procedures of whole-mount immuno-labelling protocols closely resemble classical histological immuno-staining. However, one major limiting factor for in toto immuno-staining is the penetration of antibodies, which needs to be reconciled with the molecular integrity of the tissue. Espinoza et al. proposed an initial protocol which consisted in methanol de-hydration coupled to multiple freeze-thawing steps for tissue permeabilisation [23]. This protocol reduced whole-mount immuno-labeling to under a week, but was strictly applied to small mouse embryos (E10.5).

To further optimize the quality as well as the diversity of tissue types for immuno-labelling, Renier et al. proposed the immunolabeling-enabled 3DISCO protocol (iDISCO). To increase tissue permeabilisation as well as to minimize laser absorption from heme-rich tissues (hematomas), Renier et al. carry out a “bleaching” pre-treatment consisting of hydrogen peroxide in methanol (to avoid tissue damage). To further permeabilise the tissues, the authors subject them to an overnight incubation in dimethyl sulfoxide (DMSO). To increase the signal-to-noise ratio the samples are then treated with heparin and glycine. Initially coupled to 3DISCO clearing, Renier et al. have since coupled the iDISCO protocol to the iDISCO+ clearing protocol to prevent morphological damages to the tissue during clearing. The combined use of methanol and dichloromethane is aimed at permeabilizing lipid-rich adult tissues, containing complex lipids that are not soluble in the low concentrations of detergents used in classical immuno-histochemistry protocols. However, this comes with a catch 22 situation. Indeed, the iDISCO+ clearing protocol enables a greater diffusion of the IgGs in adult tissues (especially brains), but the use of methanol restricts the repertoire of antibodies compatible for immuno-labelling.

Belle et al. have since proposed an alternate immuno-labeling protocol amenable to diverse tissue types that circumvent the tedious pre-treatment steps of the iDISCO protocol [24, 25]. We propose a simple/easy-to-implement protocol that only consists of blocking buffer in combination with a detergent, saponin. The strength of using saponin is that it is a mild non-ionic surfactant with reversible permeabilising effect. Whilst currently only used in the antibody solutions, saponin could be supplemented in all incubation steps to further increase the quality of immuno-labeling. This protocol provides several advantages to the iDISCO protocol. Firstly, by removing methanol treatments, the adaptability of this immuno-labeling protocol can be applied to a wider array of antibodies. Furthermore, by removing treatments with reagents such as heparin and DMSO, the endogenous epitopes are more stable. To date, we, and others, have combined this immuno-labeling protocol to a vast diversity of tissue types and species (mouse, primate, human, xenopus, zebrafish, other mammals and birds) [24–29]. We have currently tested 102 antibodies, 64 of which have been validated and 38 that have not worked (unpublished). Currently, the specificity in epitope labeling is very diverse, ranging from trans-membrane proteins to transcription factors. Thus, we cannot yet discuss on epitope-specific limitations of our immuno-labelling protocol to clearing protocols. That being said, out of the 38 antibodies that did not work, several may be incompatible to our immuno-labeling protocol. For instance, we see that while some antibodies did not work, incubations at 24–25 °C instead of 37 °C could successfully label the samples.

All immuno-labeling protocols listed above are amenable to every solvent-based tissue clearing (Table 1). However, we suggest that when dealing with the Belle et al. immuno-labeling protocol, one should carry out the iDISCO+ clearing protocol only on large samples (such as an adult mouse brain). When working with small samples (such as mouse embryos) we suggest using the 3DISCO clearing protocol, by-passing the lengthy iDISCO+ pre-treatments that are unnecessary in small sample sizes and could potentially disrupt endogenous epitopes.

Although an incredible push has been made to apply immuno-labelling to tissue clearing, the duration of incubation remains relatively slow (Table 1). Furthermore, there is a lack of studies attempting to answer questions relating to antibody penetration patterns in 3D. To this end, Li et al. proposed to apply a linear current (<30 V) to aid in antibody penetration [21]. Indeed, the authors report a 800-fold increase in penetration when compared to simply diffusing antibodies (staining a 4 mm-thick brain section within 30 min). Whilst this approach brings a wave of excitement, it needs to be taken with caution. Indeed, the authors reported that a net temperature increase was observed in the tissue by applying the current, which could alter epitope conformation. In addition, the tissue itself possesses a net charge and thus will also be altered by the electric current thereby altering its morphology. Further studies must focus on specific conditions required to increase antibody labeling without perturbing the tissue morphology. Recently, Kim et al. suggested that by exposing the tissue to a circular electric field rather than linear one, they could increase antibody penetration without perturbing the tissue [30]. This protocol rests on the idea that the freely moving charged antibodies would be subject to a greater displacement than the cross-linked charged proteins of the tissue. The strength in this model is that it could be coupled to other models of circular forces other than electrical. For instance, several groups have already proposed other models of forces for molecular targeting. The ACT-PRESTO protocol makes use of hydrodynamic pressure generated from a centrifugal rotation to immuno-label tissues [31]. Furthermore, one could potentially propose a magnetic force for molecular targeting.

An interesting perspective for immuno-labeling efficiency might come from the development of conjugated nanobodies, which possess lower molecular sizes (14KDa) and will bypass secondary antibody labeling. Although theoretically possible, it remains to be fully tested whether this would be compatible with clearing protocols, such as 3DISCO or CLARITY.

### Endogenous fluorescence

Advances in molecular biology brought with it the era of transgenic mouse lines. This powerful tool allowed researchers for the first time to explore in vivo a diverse range of previously validate in vitro results. One of these specific tools was the use of reporters (GFP, YFP, mCherry) to observe and locate in vivo the distribution of a specific protein. With this approach, researchers could by-pass immuno-cytochemistry to label specifically a protein. A huge advantage of tissue clearing brought the potential to visualize the expression of reporter lines in 3 dimensions. With this came the question of its compatibility with clearing protocols. Indeed, this would dramatically increase the rate at which we could visualize a specific protein without the need for time-consuming sectioning techniques.

However, this task became more complicated than anticipated. Indeed, upon the first test of tissue clearing using the BABB protocol, Dodt et al. quickly realized that the endogenous fluorescence in *Thy1-eYFP* mice, although initially present, rapidly quenched (within a few hours) [7]. Presumably, the stability of the endogenous fluorescent proteins may be altered following clearing as a result of multiple factors, such as de-hydration and pH alterations. Indeed, residual water must be present for the fluorescence to persist, although this remains to be fully studied and shown.

The first protocol that offered a more stable visualization of endogenous fluorescence came with the 3DISCO protocol. Ertürk et al. could visualize: dendritic cells, macrophages, B cells, microglia, neurons, and astrocytes in the spinal cord, spleens, and lymph nodes of transgenic mice expressing *MHCII-eGFP*, *CD11c-Venus*, *CX3CR1-eGFP*, *Thy1-eYFP*, and *hGFAP-eCFP* respectively [8, 9]. Though more stable than the BABB clearing protocol, endogenous fluorescence dissipates within 2 days [9] or in cases of lower GFP expression up to 16 h (unpublished data). More recently, Pan et al. showed that the solvent-based uDISCO tissue clearing protocol maintained endogenous fluorescence for up to 4 weeks in *Thy1-eYFP*, *CX3CR1–eGFP*, and *β-actin-eGFP* mice [12] (Table 1). In addition to analyzing GFP expression in the brain, the authors demonstrate they maintain endogenous fluorescence in multiple tissue types, including bones.

The development of CLARITY-based clearing protocols led to other protocols for the study of endogenous fluorescence. Indeed, using *Thy1-eYFP* mice, Chung et al. successfully observed GFP-expressing axons in adult mouse brains following clearing [15, 16]. More recently, *Ye* et al. have successfully maintained endogenous fluorescence of brains from the *Arc-TdTomato* mouse trap line [19]. However, It remains relatively unclear the length of time CLARITY-cleared samples can maintain endogenous fluorescence. Furthermore, although CLARITY-based protocols maintain GFP/RFP expression they have only been tested using the *Arc-TdTomato* or the *Thy1-eYFP* mouse lines, both of which are notoriously known for expressing high levels of fluorescence.

Recently, a novel solvent-based clearing protocol using ethyl cinnamate (Eci) was proposed by Klingberg et al. [32]. The authors are able to clear various tissues types and maintain endogenous fluorescence in *Cd11c-eYFP* mice for up to 14 days. The de-hydration steps are done in ethanol and can be combined to all solvent-based imaging media, such as BABB and DBE. In regards to its toxicity, similarly to DBE, Eci is also a Food and Drug Administration-approved compound. However, since the de-hydration steps are carried out in ethanol, the transparency attained by this protocol remains to be fully evaluated.

Currently, the most successful clearing protocols for maintaining endogenous fluorescence remain the SeeDB 2 (See Deep Brain) and CUBIC protocols, which rely on fructose and sucrose/triethanol amine solutions respectively [33, 34]. The main limitation with this procedure is the size of samples processed. Indeed, as of now, only small specimens or tissue slices have been successfully cleared using SeeDB or SeeDB 2. Moreover, samples are incompatible to macromolecule labeling and hence cannot be coupled to immuno-histochemistry [35].

An alternative for visualizing proteins of interest following tissue clearing is to package a reporter construct into a high-titer adeno-associated virus (AAV). In recent years, both aqueous and solvent-based clearing protocols have adapted the use of viral reporter protein expression to their protocols. *Pan* et al. have successfully traced motor neuron projections using a viral infection of AAV2-Synapsin-GFP/RFP construct [12]. On the other hand, the compatibility of AAV and rabies-virus tracing to the CLARITY protocol have also been successful to label distinct neuronal populations or to distinguish neuronal activity [19, 36, 37].

Collectively, the study of endogenous fluorescence with CLARITY and 3DISCO-related protocols remain largely to be studied. Of note, whilst several protocols have successfully maintained endogenous fluorescence none have compared the fluorescent signal levels to traditional 2D Immuno-histochemistry preparations. As a result, these studies may be underestimating the endogenous fluorescence expression patterns within the sample. Effort needs to be invested in testing supplemental transgenic reporter lines with varying expression levels in order to attest the full potential of clearing protocols in maintaining endogenous fluorescence. Importantly, an alternative strategy to by-pass endogenous fluorescence stability is by using antibodies targeted against the endogenous fluorescent proteins (GFP, YFP, mCherry) [24, 38].

### The world of RNA

Until recently, clearing protocols have focused on targeting proteins, either using macromolecules such as antibodies or using transgenic mice expressing reporters. However, it has been known for some time that whole-mount in situ hybridization works relatively well. Still, most clearing protocol have not yet adapted to this approach. Yet, a wealth of information is waiting to be seized with acquiring molecular information of cells using in situ probes such as smFISH techniques.

Early reports of in situ hybridization compatible to the PACT clearing protocol were initially reported on the abundantly expressed RNA, b-actin [18]. Whilst promising, this study focused on 100 μm-thick tissue sections. Recently, the group of Deisseroth provided an optimized CLARITY protocol, compatible with smFISH labeling following clearing [39]. Sylwestrak et al. propose an improved protocol for in situ hybridization increasing cross-linking of hydrogel amine-containing monomers to nucleic acid arms, using 1-Ethyl- 3-3-dimethyl-aminopropyl carbodimide (EDC). They could efficiently label miRNAs and visualize their expression in 3-dimension. This study expands the sample size of study to 3 mm-thick brain blocks.

Thus far, no studies have proved the adaptability of solvent-based clearing protocols to mRNA analysis. However, methanol is sometimes used for whole-mount in situ hybridization and thus the iDISCO+ protocol could likely be compatible to RNA analysis. Other solvent-based protocols, such as 3DISCO and uDISCO, are yet to be studied.

## Handling cleared tissues: From physical to software limitations

### The “physicality” of cleared samples

Before choosing a specific clearing protocol, one should assess the “physical” components of their specific tissue. Indeed, tissues are very heterogeneous and therefore will respond differently to each clearing protocol. As listed in Table 1, the transparency reached by each clearing protocol will depend on the tissue studied. For these reasons, the following section will focus on the effect of size, handling, and further processing of tissues following solvent-based and aqueous-based clearing.

One of the critical differences between clearing protocols is the size alteration of the samples (Table 1). Whilst some aqueous-based clearing protocols have reported little-to-no size alterations, the solvent-based clearing protocols differ in that respect. CLARITY and its derivatives have shown a dynamic alteration in size, with an initial swelling of the sample, which can reduce to non-significant differences depending on the imaging medium used [17]. On the other hand, tissues cleared with 3DISCO lead to tissue shrinkage between 30 and 50% depending on tissues (with a higher shrinkage in water-rich tissues). Following methanol clearing, using the iDISCO+ protocol, tissue-shrinkage reduces modestly (About 10% in Methanol/H_2_O, higher in Methanol/PBS). In addition to maintaining size, methanol-cleared tissues remain “softer”. The advantage of this allows further tissue processing. However, the disadvantage is that the tissue remains more fragile than with 3DISCO-cleared samples.

Following clearing, tissues must be observed either by light-sheet, confocal, or multi-photon microscopy. However, the issue of size really comes into play when acquiring images using a light-sheet microscope. Indeed, the strength of this tool is to image large samples with little acquisition time. Whilst initially limited by the commercially available platforms, the size of samples that can be placed into the imaging reservoir has increased owing to the development of homemade platforms. However, one is restricted in regards to the size of the sample by the working distance of the lens used. As a result, 3DISCO and uDISCO-cleared samples have an advantage over other methods such as the iDISCO+ and CLARITY. For instance, using the 3DISCO protocol, our group and others have reported successful clearing and imaging of entire human embryos and fetuses without the need for dissection [25, 40]. 3DISCO-cleared samples result in anisotropic shrinkage and therefore are incompatible with template-based registration algorithms, such as ClearMap. It remains to be studied whether uDISCO-cleared samples could be registered [11].

## What the future holds

With the field only being in its infancy, one can only predict a bright future for the field of tissue clearing. Over the past decade, tissue clearing strategies have become more attuned to the ever-expending demands of cellular and molecular biology research, allowing laboratories to no longer choose between breadth and depth when analyzing a said sample.

One major potential of tissue clearing strategies is their adaptability to multiple species. Whilst several groups have successfully applied tissue clearing protocols to post-mortem human tissue sections, the full extent of human tissue applicability to clearing has not yet been studied. Undeniably, the study of human development has been a key topic of interest for many scientists and has for a century relied on 2D tissue sections of paraffin embedded embryos. Until recently, modern embryology textbooks still portrayed many of these representations. To address this, using 3DISCO and iDISCO+ clearing, we were able to successfully validate over 40 different antibodies and image entire human embryos and fetuses in 3D at cellular resolution [25]. Our findings provide insights into the specificities of human peripheral nervous system, vascular, cardiopulmonary, urogenital, and muscular development. We have elaborated a repository to harbor around 1000 acquisitions of 36 human embryos and fetuses which can be visited here: https://transparent-human-embryo.com/. Our vision is a collaborative effort of several laboratories to expand this repository into a 3D atlas for the study of human development. Tissue clearing for the study of human development is only beginning, Casoni et al. have already shed light on the advantages of using tissue clearing to study the development of gonadotropin-releasing hormone neurons in the hypothalamus of human embryos [40]. These novel studies allow for the first time an in-depth molecular analysis of human development and thus will certainly shed new light on pathological developments of human embryos and fetuses.

A further application of tissue clearing has become critical in the field of oncology. Until recently, little was known about the three-dimensional complexity of tumors in many cancers. Clinically, diagnoses have for centuries relied on 2-dimensional analysis of sections from biopsied tumors. Such techniques limit pathologists to a snapshot of the tumor. Furthermore, artifacts linked to tissue processing such as sectioning may impede the evaluation of the biopsied specimen. Since, developments of imaging tools such as X-ray computed tomography or optical coherence tomography have provided better 3D characterization of tumor but still provided poor resolution. To address these limitations, Torres et al. used BABB clearing on kidney, breast, prostate and liver biopsies followed by multi-photon microscopy [41]. The authors reported advantages such as the analysis of low-grade abnormalities in cellular growth, neoplasia, as well as a stronger detection of tumor invasion within the tissue. In addition, Van Royen et al. using BABB clearing could further apply immuno-histochemistry to cleared prostate biopsies followed by confocal microscopy. Of interest, the authors note that the cleared specimens retained a high DNA yield and could further be subjected to a polymerase chain reaction [42]. The extent of tissue clearing techniques application towards the field of cancer is not limited to its clinical applications. More particularly in the field of breast cancer, several laboratories have recently begun to apply these techniques for the cellular and molecular analysis of mammary gland development as well as mammary glands dysplasia. Davis et al. recently described using CUBIC and SeeDB clearing the clonal analysis of mammary glands [43]. Furthermore, Lloyd-Lewis et al. further described the use of multiple clearing protocols on mammary gland clearing [44]. Finally, one exciting avenue for tissue clearing strategies will be the unbiased observation of entire tissues following drug administration. Such applications have already been shown on whole-brains of mice treated with antipsychotic drugs, haloperidol [11].

However, acquisition of such large raw data comes with a cost. For instance, acquiring an entire adult mouse brain can go up to 2 terabytes of data, depending on the machine used, objectives, resolution, and magnification. This number increases exponentially if this acquisition is tiled or if multiple channels of emission are acquired. As a result, the bottleneck in the field of tissue clearing is fast-becoming how to handle this complex data set. Many of the existing compression software available poorly answer the need of light-sheet acquisitions of cleared tissues; this is mostly due to the size and complexity of the data set. Though several laboratories are beginning to make ground, research will need to expand in to develop novel compression formats [45].

Taken together, whilst tissue clearing protocols have already began to shatter many of our common understandings of how tissues are organized in 3D, it is without a doubt that its applications in new fields of research further change our understanding of function in health and disease and will become a key component of cellular and molecular biology techniques in laboratories across the globe.

## Abbreviations

3DISCO: 3D imaging of solvent-cleared organs
AAV: Adeno-Associated Virus
BABB: Benzyl Alcohol/Benzyl Benzoate
CLARITY: Clear Lipid-exchanged Acrylamide-hybridized Rigid Imaging / immunostaining / in situ hybridization-compatible Tissue hYdrogel
DBE: Dibenzyl ether
DCM: Dichloromethane
DPE: Di-phenyl ether
Eci: Ethyl cinnamate
EDC: 1-Ethyl- 3-3-dimethyl-aminopropyl carbodimide
iDISCO: Immunolabeling-enabled 3DISCO protocol
iDISCO_+_: Immunolabeling-enabled three-dimensional imaging of solvent-cleared organs
LSFM: Light sheet fluorescence microscopy
PACT: Passive CLARITY technique
PARS: Perfusion assisted agent release in situ
SeeDB: See deep brain
smFISH: small-molecule fluorescent in situ hybridization
THF: Tetrahydrofuran
uDISCO: ultimate-3DISCO
